# Incentive delivery timing and follow-up survey completion in a prospective cohort study of injured children: a randomized experiment comparing prepaid and postpaid incentives

**DOI:** 10.1186/s12874-021-01421-8

**Published:** 2021-10-27

**Authors:** Morgan M. Millar, Lenora M. Olson, John M. VanBuren, Rachel Richards, Murray M. Pollack, Richard Holubkov, Robert A. Berg, Joseph A. Carcillo, Patrick S. McQuillen, Kathleen L. Meert, Peter M. Mourani, Randall S. Burd

**Affiliations:** 1 Department of Internal Medicine, University of Utah School of Medicine, 295 Chipeta Way, Salt Lake City, UT 84132 USA; 2 Department of Pediatrics, University of Utah School of Medicine, 295 Chipeta Way, Salt Lake City, UT 84108 USA; 3 Department of Pediatrics, Children’s National Health System and the George Washington University School of Medicine and Health Sciences, Washington, DC USA; 4 Department of Anesthesiology and Critical Care Medicine, Children’s Hospital of Philadelphia, Philadelphia, PA USA; 5 Department of Critical Care Medicine and Pediatrics, Children’s Hospital of Pittsburgh, Pittsburgh, PA USA; 6 Department of Pediatrics, Benioff Children’s Hospital, University of California San Francisco, San Francisco, CA USA; 7 Department of Pediatrics, Children’s Hospital of Michigan, 3901 Beaubien, Detroit, MI 48201 and, Central Michigan University, Mt. Pleasant, MI USA; 8 Central Michigan University, Mt. Pleasant, MI USA; 9 Department of Pediatrics, Children’s Hospital Colorado and University of Colorado School of Medicine, Aurora, CO USA; 10 Division of Trauma and Burn Surgery, Children’s National Medical Center, 111 Michigan Ave NW, Washington, DC 20010 USA

**Keywords:** Cohort studies, Surveys and questionnaires, Methods, Motivation, Patient selection, Random allocation

## Abstract

**Background:**

Retaining participants over time is a frequent challenge in research studies evaluating long-term health outcomes. This study’s objective was to compare the impact of prepaid and postpaid incentives on response to a six-month follow-up survey.

**Methods:**

We conducted an experiment to compare response between participants randomized to receive either prepaid or postpaid cash card incentives within a multisite study of children under 15 years in age who were hospitalized for a serious, severe, or critical injury. Participants were parents or guardians of enrolled children. The primary outcome was survey response. We also examined whether demographic characteristics were associated with response and if incentive timing influenced the relationship between demographic characteristics and response. We evaluated whether incentive timing was associated with the number of calls needed for contact.

**Results:**

The study enrolled 427 children, and parents of 420 children were included in this analysis. Follow-up survey response did not differ according to the assigned treatment arm, with the percentage of parents responding to the survey being 68.1% for the prepaid incentive and 66.7% with the postpaid incentive. Likelihood of response varied by demographics. Spanish-speaking parents and parents with lower income and lower educational attainment were less likely to respond. Parents of Hispanic/Latino children and children with Medicaid insurance were also less likely to respond. We found no relationship between the assigned incentive treatment and the demographics of respondents compared to non-respondents.

**Conclusions:**

Prepaid and postpaid incentives can obtain similar participation in longitudinal pediatric critical care outcomes research. Incentives alone do not ensure retention of all demographic subgroups. Strategies for improving representation of hard-to-reach populations are needed to address health disparities and ensure the generalizability of studies using these results.

**Supplementary Information:**

The online version contains supplementary material available at 10.1186/s12874-021-01421-8.

## Introduction

Prospective studies evaluating health outcomes over time depend on successful completion of follow-up assessments by enrolled participants. Longitudinal studies that rely on recontacting participants to collect additional data require more from participants than one-time cross-sectional studies. Repeated outcome measurement places an additional burden on participants and may contribute to higher levels of non-response [1, 2]. Minimizing non-response at follow-up is especially critical for ensuring continued representation of enrolled participants from hard-to-reach populations. Minimizing participant attrition in longitudinal studies ensures statistical power, demographic representation, and preserves study validity and integrity [3–8].

Incentives are frequently used to encourage participants to complete follow-up assessments. Previous studies have evaluated how strategies such as incentives and contact methods affect retention in health and epidemiological research, including cohort studies and randomized trials [9–12]. Findings from this research suggest that monetary incentives are effective, but nonmonetary incentives are not [9]. Additionally, increasing the dollar amount of incentives has improved retention [10]. Systematic reviews have shown that monetary incentives improve retention in randomized trials [9, 13] and prospective cohort studies [11].

Although types of incentive and incentive amounts have been studied, minimal attention has been given to incentive timing, i.e., whether incentives are postpaid or prepaid, in health outcomes research. Prepaid incentives are commonly used by social scientists and public opinion researchers. Prepaid incentives resulted in higher response rates than the promise of a postpaid incentives in cross-sectional [14–17] and longitudinal [18–20] survey research. It is less clear whether prepaid incentives are similarly effective in health outcomes studies. In prospective clinical research, postpaid incentives are more commonly used. One study that compared the effect of prepaid and postpaid incentives on retention in a randomized trial found inconsistent effects [21]. Few investigators have examined whether prepaid incentives are more effective at retaining cohort participants for follow-up data collection [12]. The existing literature offers no conclusive guidance on whether prepaid incentives improve retention in health outcomes research.

Survey researchers have also examined whether incentive timing influences the demographic composition of respondents. In some cases, prepaid incentives improved demographic representation in cross sectional surveys [22, 23]. In other instances, prepaid incentives skewed the representativeness of participants [24, 25]. Demographic characteristics such as lower socioeconomic status have been associated with higher attrition in prior cohort studies [26]. These studies support the need to evaluate the impact of incentive timing on the demographics of retained participants in prospective health research. Prepaid incentives may also encourage faster response than postpaid incentives, which can shorten the data collection period or reduce the number of contact attempts [27, 28]. Outcomes such as response speed have received less attention than response rates. Factors affecting response speed in longitudinal health outcomes research have not been examined.

The purpose of this study was to determine whether prepaid incentives result in higher retention than postpaid incentives in a longitudinal study of injured children. We conducted a randomized experiment to evaluate the timing of incentive delivery on parents’ response to a six-month follow-up survey. We hypothesized that a prepaid incentive would result in a higher survey response rate compared to the promise of a postpaid incentive. A secondary aim examined whether a prepaid incentive would reduce the required number of contact attempts needed to reach participants. We also assessed whether parent and child demographic characteristics were associated with retention and evaluated whether prepaid incentives aid in the retention of a demographically representative sample.

## Materials and methods

### Study design and participants

We conducted a parallel, 1:1 randomized trial (“experiment”) nested within a prospective cohort study (“cohort study”) to assess the effect of incentive timing on participant response to a follow-up survey. This experiment was embedded within the “Assessment of Functional Outcomes and Health-Related Quality of Life after Pediatric Trauma Study.” The aim of this study was to identify factors associated with injured children’s functional status at hospital discharge, and the relationship between functional status at discharge with six-month functional status and health-related quality of life. Functional status—the ability to perform activities of daily living—measure six domains: mental status, sensory, communication, motor, feeding, and respiratory function [29]. The cohort study was conducted at seven sites in the United States from March 2018 to February 2020. The Institutional Review Board at the University of Utah approved this study through a central mechanism (Approval #00105435). Additional details about the children’s injuries and the survey measures used in this study have been previously reported [30].

Children under 15 years in age who were treated for a serious, severe, or critical injury to one or more major body regions (head, thorax, abdomen, spine, or extremity) were eligible for enrollment in the cohort study. Patients with major burn injuries were excluded, as were children whose parents or guardians (hereafter referred to as parents) did not speak English or Spanish. Eligible children were enrolled at seven hospital sites, all level 1 pediatric trauma centers within the National Institutes of Health-funded Collaborative Pediatric Critical Care Research Network (CPCCRN). The participant of interest for this experimental study was the parent who provided consent for the child’s participation. Parents received all follow-up communications, were asked to complete the survey, and were assigned to an experimental arm.

### Recruitment, follow-up protocol, and randomization

At each hospital (study site), research coordinators reviewed the daily census to identify eligible children. The cohort study sampling strategy was designed to promote equal enrollment of patients with isolated injuries in each body region. We set a goal to enroll 50 patients per study site per year, with 70% comprised of children with one injured body region and 30% children with multiple injured body regions. Every three months, we adjusted enrollment across sites to ensure enrollment in each category. The original goal was to enroll up to 840 patients into the cohort study. Statistical power was calculated to detect a difference in the proportion of parents who completed the survey between experimental arms. We initially planned to perform interim monitoring of experimental results after 210, 420, and 630 participants had completed follow-up. If interim analyses indicated one incentive type to be superior to the other, we planned to stop randomization and proceed with the superior method for subsequently enrolled participants. The cohort study ended early due to funding after enrolling 427 children.

Research coordinators obtained written informed consent and collected baseline data using medical records and standardized questionnaires administered to parents at discharge. Six months after hospital discharge, the parent who signed the consent form was asked to complete a telephone survey about the child’s current functional status and health-related quality of life. The Pediatrics Clinical Trials Office at the University of Utah made all contacts associated with the follow-up survey. These contacts consisted of a reminder letter at three months, a second reminder letter one week before the telephone survey, and a text message reminder one day before the telephone survey. To collect the survey data, at least three telephone call attempts were made on different days and times of day according to preferences parents specified at enrollment. If these attempts were unsuccessful, the study team attempted to reach a designated alternate contact to confirm the parent’s availability and contact information. If these contacts were also unsuccessful, the parent was emailed a link to an abbreviated web version of the survey two weeks after the last call attempt. If the parent did not respond to the web survey within another two weeks, medical records were reviewed to determine if six-month outcomes could be assessed from this source.

The contact protocol and materials for both experimental arms were the same except for the six-month reminder letter, which contained the intervention. This letter was either accompanied by a cash card (prepaid incentive arm) or informed the parent that a cash card would be sent after survey completion (postpaid incentive arm). After enrollment, parents were randomly assigned to one of the two incentive arms. We stratified incentive assignments within each study site and by injury type using a pre-generated randomization sequence that was created by the study biostatistician using statistical software. The sequence was concealed from all study staff except for the central research coordinator overseeing incentive delivery. The randomization sequence was reset daily by IT staff and parents were automatically assigned to a study arm as they enrolled in the study using the REDCap (Research Electronic Data Capture) platform [31]. The study site that enrolled the participant did not know which incentive arm was assigned. Because they participated in the contact protocols and delivery of the incentives, the interviewers who administered the surveys were aware of parents’ incentive assignments.

### Intervention, outcomes, and additional variables

The experimental intervention was incentive timing, categorized as a prepaid or postpaid incentive. Parents either (1) received a US$50 cash card in advance of the follow-up survey or (2) were informed that they would receive the cash card after completing the survey. Similar to a debit card, the cash card could be used at any retailer.

The primary outcome of this experiment was six-month survey completion, categorized as completed or not completed. A telephone survey response was classified as complete if sufficient information was obtained for scoring at least four of the five instruments included in the survey. The web survey was considered complete if containing enough information for scoring three out of the four instruments. A survey was classified as not completed if the parent did not respond to any of the survey requests or did not complete enough items to reach the completion thresholds. The secondary outcome was the number of call attempts needed to reach participants, as recorded by the interviewers making the telephone calls.

Another secondary aim was to assess whether demographic characteristics were associated with the primary outcome of survey completion. Available demographic variables included parental educational attainment and the parent’s preferred language (English or Spanish), household income and household size, and the race, ethnicity, age, sex, and insurance status of the child. These variables were obtained from a baseline survey.

### Statistical analysis

We calculated descriptive statistics for patient and parent demographics by each experimental arm and within the entire sample. Categorical measures were summarized with counts and percentages. Continuous measures were summarized using medians and the 25^th^ and 75^th^ percentile values to account for non-normal distributions. We calculated the proportion of parents who completed the survey by experimental arm. To test for differences in survey completion by experimental arm, we performed logistic regression models predicting survey completion, controlling for incentive assignment and study site. To test for demographic differences in survey completion, we performed logistic regression models predicting survey completion, controlling for the demographic variable and study site. In a supplemental analysis, we assessed the effect of the interaction of each demographic variable with incentive type on likelihood of survey response. Significance was defined at *p*<0.05. Analyses were performed using SAS version 9.4 (Cary, NC).

## Results

Across all study sites, 835 children were assessed for eligibility in the cohort study, 654 met inclusion criteria, 493 were approached for consent, and 428 were consented to participate (See Flow Diagram, Additional file 1). One patient was excluded due to the absence of a qualifying injury, resulting in a final sample of 427 children with their consenting parents. To evaluate the effect of incentive timing, we limited the analyses to the 420 parents who were randomly assigned to an experimental arm. The 420 randomized parents were analyzed according to their originally assigned treatment arm (prepaid incentive n=204, postpaid incentive n=216).

Most parents reported a household size of three or four individuals (54.5%; Table 1). The largest share of parents had a high school education or less (35.7%) and 15.7% reported an annual household income of less than $15,000. Only 3.6% of parents were surveyed in Spanish. The median age of the injured children was 7.2, 36.9% were female, 11.2% were Hispanic, and 64.8% were white. A summary of the children’s injury characteristics is included in Additional file 2.

**Table 1:** Demographic characteristics of children in the study sample, overall and by experimental arm

	Incentive timing		
	Before survey (*N* = 204) N (%)	After survey (*N* = 216) N (%)	Overall (*N* = 420) N (%)
Age in years^a^	6.6 [2.9, 11.4]	8.3 [2.5, 11.9]	7.2 [2.6, 11.7]
Female	77 (37.7)	78 (36.1)	155 (36.9)
Race			
American Indian or Alaska Native	1 (0.5)	1 (0.5)	2 (0.5)
Asian	2 (1.0)	5 (2.3)	7 (1.7)
Black or African American	40 (19.6)	54 (25.0)	94 (22.4)
White	149 (73.0)	123 (56.9)	272 (64.8)
More than one	10 (4.9)	26 (12.0)	36 (8.6)
Other	2 (1.0)	6 (2.8)	8 (1.9)
Unknown	0 (0.0)	1 (0.5)	1 (0.2)
Ethnicity			
Hispanic or Latino	22 (10.8)	25 (11.6)	47 (11.2)
Not Hispanic or Latino	180 (88.2)	191 (88.4)	371 (88.3)
Unknown	2 (1.0)	0 (0.0)	2 (0.5)
Parent preferred language-Spanish	8 (3.9)	7 (3.2)	15 (3.6)
Insurance			
Private/commercial	94 (46.1)	93 (43.1)	187 (44.5)
Medicaid/Medicare	89 (43.6)	105 (48.6)	194 (46.2)
Self-Pay/no insurance	9 (4.4)	3 (1.4)	12 (2.9)
More than one/other	10 (4.9)	13 (6.0)	23 (5.5)
Unknown	2 (1.0)	2 (0.9)	4 (1.0)
Annual household income			
Less than $15,000	32 (15.7)	34 (15.7)	66 (15.7)
$15,000-$19,999	8 (3.9)	13 (6.0)	21 (5.0)
$20,000-$29,999	15 (7.4)	23 (10.6)	38 (9.0)
$30,000-$39,999	12 (5.9)	21 (9.7)	33 (7.9)
$40,000-$49,999	18 (8.8)	19 (8.8)	37 (8.8)
$50,000-$74,999	25 (12.3)	26 (12.0)	51 (12.1)
≥ $75,000	73 (35.8)	69 (31.9)	142 (33.8)
Unknown	21 (10.3)	11 (5.1)	32 (7.6)
Household size			
2	8 (3.9)	11 (5.1)	19 (4.5)
3-4	110 (53.9)	119 (55.1)	229 (54.5)
5 or more	83 (40.7)	81 (37.5)	164 (39.0)
Unknown	3 (1.5)	5 (2.3)	8 (1.9)
Primary caregiver education			
High school/GED or less	70 (34.3)	80 (37.0)	150 (35.7)
Associates/vocational degree/some college	46 (22.5)	65 (30.1)	111 (26.4)
Bachelor’s degree	40 (19.6)	41 (19.0)	81 (19.3)
Graduate degree	37 (18.1)	24 (11.1)	61 (14.5)
Unknown	11 (5.4)	6 (2.8)	17 (4.0)

^a^Summarized as median [25^th^ percentile, 75^th^ percentile]

We obtained survey responses from 67.4% (283/420) of the parents. Survey completion did not differ based on incentive timing. The response rate for the prepaid incentive was 68.1%, and 66.7% for the postpaid incentive (*p*=0.61, Table 2). A median of two telephone calls was associated with successful contact with the parent. The number of telephone calls needed to reach parents also did not differ based on when the incentive was provided (*p*=0.22). Regardless of the incentive offered, the highest percentage of parents were reached on the first call (39.1% for the prepaid incentive, 49.6% for the postpaid incentive).

**Table 2:** The effect of incentive timing on six-month follow-up survey completion outcomes in a prospective cohort study

	Incentive timing			
	Before survey (*N* = 204) N (%)	After survey (*N* = 216) N (%)	Overall (*N* = 420) N (%)	*P*-value^1^
**Response outcome:**				
Follow-up survey completed				0.61
No	65 (31.9)	72 (33.3)	137 (32.6)	
Yes	139 (68.1)	144 (66.7)	283 (67.4)	
No. of calls required for contact				0.22
1	43 (39.1)	62 (49.6)	105 (44.7)	
2	31 (28.2)	26 (20.8)	57 (24.3)	
3	17 (15.5)	15 (12.0)	32 (13.6)	
4	8 (7.3)	8 (6.4)	16 (6.8)	
5	5 (4.5)	4 (3.2)	9 (3.8)	
6	3 (2.7)	7 (5.6)	10 (4.3)	
7	1 (0.9)	2 (1.6)	3 (1.3)	
8	2 (1.8)	0 (0.0)	2 (0.9)	
Unknown^2^	0 (0.0)	1 (0.8)	1 (0.4)	

^1^*P*-value associated with incentive assignment obtained from logistic regression models predicting each individual variable, with incentive assignment and study site as predictors

^2^Not included in *p*-value calculation

Survey response varied by demographic characteristics (Table 3). Hispanic or Latino children comprised 6.4% of respondents and 21.2% of non-respondents (*p*<0.001). Spanish-speaking parents were less likely than English speakers to complete the survey (0.4% of respondents compared to 10.2% of non-respondents; *p*<0.001). Children with Medicaid were less likely to be represented among the responses (37.8% of respondents compared to 63.5% of non-respondents; *p*<0.001). Children whose parents had a high school diploma or less education were under-represented among respondents (27.6%) compared to non-respondents (52.6%; *p*<0.001). Fewer respondents than non-respondents were from families earning less than $15,000 per year (11.7% vs. 24.1%; *p*<0.001). Parents reporting household sizes of five or more individuals were underrepresented among respondents (34.6% of respondents compared to 48.2% of non-respondents; *p*<0.013). The interaction of each demographic variable with incentive assignment did not predict on survey completion (Additional file 3). Completion patterns by demographics were similar regardless of incentive timing.

**Table 3:** Follow-up survey completion by child and parent demographic characteristics

	Follow-up survey completed			
	No (*N* = 137) N (%)	Yes (*N* = 283) N (%)	Overall (*N* = 420) N (%)	*P*-value^1^
Age in years^2^	5.9 [2.2, 11.6]	7.8 [2.7, 11.7]	7.2 [2.6, 11.7]	0.47
Sex				0.31
Male	81 (59.1)	184 (65.0)	265 (63.1)	
Female	56 (40.9)	99 (35.0)	155 (36.9)	
Race				0.101
American Indian or Alaska Native	1 (0.7)	1 (0.4)	2 (0.5)	
Asian	2 (1.5)	5 (1.8)	7 (1.7)	
Black or African American	40 (29.2)	54 (19.1)	94 (22.4)	
White	76 (55.5)	196 (69.3)	272 (64.8)	
More than one	13 (9.5)	23 (8.1)	36 (8.6)	
Other	5 (3.6)	3 (1.1)	8 (1.9)	
Unknown^3^	0 (0.0)	1 (0.4)	1 (0.2)	
Ethnicity				<.001
Hispanic or Latino	29 (21.2)	18 (6.4)	47 (11.2)	
Not Hispanic or Latino	108 (78.8)	263 (92.9)	371 (88.3)	
Unknown^3^	0 (0.0)	2 (0.7)	2 (0.5)	
Parent preferred language				<.001
English	123 (89.8)	282 (99.6)	405 (96.4)	
Spanish	14 (10.2)	1 (0.4)	15 (3.6)	
Insurance				<.001
Private/commercial	41 (29.9)	146 (51.6)	187 (44.5)	
Medicaid/Medicare	87 (63.5)	107 (37.8)	194 (46.2)	
Self-pay/no insurance	4 (2.9)	8 (2.8)	12 (2.9)	
More than one/other	4 (2.9)	19 (6.7)	23 (5.5)	
Unknown^3^	1 (0.7)	3 (1.1)	4 (1.0)	
Annual household income				<.001
Less than $15,000	33 (24.1)	33 (11.7)	66 (15.7)	
$15,000-$19,999	9 (6.6)	12 (4.2)	21 (5.0)	
$20,000-$29,999	17 (12.4)	21 (7.4)	38 (9.0)	
$30,000-$39,999	11 (8.0)	22 (7.8)	33 (7.9)	
$40,000-$49,999	16 (11.7)	21 (7.4)	37 (8.8)	
$50,000-$74,999	9 (6.6)	42 (14.8)	51 (12.1)	
≥ $75,000	27 (19.7)	115 (40.6)	142 (33.8)	
Unknown^3^	15 (10.9)	17 (6.0)	32 (7.6)	
Household size				0.013
2	7 (5.1)	12 (4.2)	19 (4.5)	
3-4	61 (44.5)	168 (59.4)	229 (54.5)	
5 or more	66 (48.2)	98 (34.6)	164 (39.0)	
Unknown^3^	3 (2.2)	5 (1.8)	8 (1.9)	
Primary caregiver education				<.001
High school/GED or less	72 (52.6)	78 (27.6)	150 (35.7)	
Associates/vocational degree/some college	32 (23.4)	79 (27.9)	111 (26.4)	
Bachelor’s degree	14 (10.2)	67 (23.7)	81 (19.3)	
Graduate degree	12 (8.8)	49 (17.3)	61 (14.5)	
Unknown^3^	7 (5.1)	10 (3.5)	17 (4.0)	

^1^*P*-value reported from logistic regression predicting follow-up completion with variable and study site as predictors

^2^Summarized as median [25^th^ percentile, 75^th^ percentile]

^3^Not included in the *p*-value calculation

## Discussion

Collecting long-term health outcomes data after discharge is time-consuming and costly. The resources and optimal approaches for conducting longitudinal follow-up are not well-defined. In this study, we evaluated whether prepaid incentives retained more parents than postpaid incentives in a study of injured children. Contrary to our hypothesis, incentive timing did not influence the likelihood of survey completion. This result departs from research showing that prepaid incentives were more effective than postpaid incentives for improving cross-sectional survey completion [15–17] and longitudinal survey retention [18–20].

Several explanations may account for our results. Evidence in support of prepaid incentives comes primarily from cross-sectional survey research [15, 17]. Incentives are used in longitudinal surveys, but less is known about the optimal use of incentives to reduce attrition in these studies [19]. Even fewer studies have addressed how incentive timing affects retention in prospective cohort studies. Incentive timing may not predict retention in longitudinal health outcomes research. Systematic reviews show that retention in health studies improves as more response-inducing strategies are incorporated [9, 11]. Strategies other than incentives include multiple reminders, varied modes of contact, and sending a second copy of a paper questionnaire to non-respondents.

The effect of incentive timing on retention may also vary based on the subject matter of a survey or across different target populations. A survey’s subject matter predicts cross-sectional survey participation [15]. Survey response is higher when questions are interesting to the participants. In the current study, we asked parents about their children’s functional status and quality of life after injury. This subject matter is relevant to parents because of the family burden associated with a child’s long-term impairment [32]. The relevance of the content may have affected survey completion more than incentive timing. In a similar study, parents of injured children expressed gratitude for follow-up calls evaluating their child’s status [33], supporting interest in this subject.

The cash card we used as an incentive could also account for our results. The effect of prepaid incentives can depend on the currency offered. A prepaid cash incentive retained more participants than a prepaid gift card in a longitudinal survey of recent high school graduates [34]. A prepaid cash card produced a lower response rate than a prepaid check in a survey of physicians [35]. The incentive dollar amount is also relevant to the timing of delivery. Small, prepaid cash incentives were associated with more responses than larger, postpaid incentives [36]. A smaller prepaid incentive could have produced different results in our study. The magnitude of an incentive’s effect on survey response also depends on the survey mode. Many studies that obtained higher response with prepaid incentives used mailed paper surveys [15, 16]. The effect of prepaid incentives on response in telephone surveys has been smaller [17].

We also assessed whether incentive timing led to differences in the demographic composition of respondents and non-respondents. Prepaid incentives improved demographic representation in some cross-sectional studies [22, 23], but decreased representation in others [24, 25]. We found no demographic differences between the respondents and non-respondents based on incentive timing. The association of incentive timing with responses and demographic representation in survey research is an evolving area of investigation [17].

We observed that some demographic characteristics were associated with follow-up completion, including child ethnicity and insurance status, household income, and parental education. Others have noted the challenge researchers have in recruiting participants from all racial and ethnic groups and diverse socioeconomic backgrounds [37]. Methods to improve participation among underrepresented populations must be tailored to address the multiple barriers faced when participating in research [38]. Prior research suggests barriers to participation include mistrust of medical research, language barriers, and demands and inconveniences of participation [38, 39]. Community-based, tailored, and personalized recruitment efforts may facilitate continued engagement with underrepresented populations [39–41]. More research is needed to identify specific strategies that ensure demographic representation in health outcomes research and ensure the generalizability of the findings of this research [42].

We anticipated that a prepaid incentive would encourage parents to answer the study’s telephone calls and reduce the need for multiple call attempts. Prepaid incentives can reduce the level of effort required to obtain follow-up responses [27, 28]. In this study, the number of calls research coordinators placed did not vary by incentive timing. We contacted most parents on the first or second attempt regardless of incentive type. Contact at the initial attempts may be related to parents’ interest in the study or the reminder letter’s impact.

Unlike prior incentive timing experiments, the respondents in our study were proxies providing information about the enrolled children. Parent-proxy reporting of children’s quality of life or other health outcomes is frequently used in pediatrics [43]. Compared to other medical specialties, pediatrics is more family-oriented, and parents play a larger role in healthcare decision-making [44]. These unique circumstances may require different retention methods than studies that use direct reports from the participant.

It is difficult to retain participants in prospective cohort studies [45]. This experiment provides guidance for designing future longitudinal studies of critically ill and injured children. Our results show that parents in longitudinal pediatric critical care studies can be retained with either a prepaid or postpaid incentive. Postpaid incentives are more commonly used in health outcomes research. In our experience, postpaid incentives are less likely to be restricted by organizational accounting policies. Postpaid incentives may be more suitable when these restrictions exist. Prepaid incentives must be carefully considered because of cost. Prepaid cash incentives are less expensive when offered in smaller amounts than postpaid incentives. Recipients of prepaid incentives delivered as a check are unlikely to cash them if not participating in the study, making them more cost-effective [46]. Prepaid incentives can also establish goodwill and trust [47]. Prepaid incentives do not require additional follow-up contact for delivery. Incentives enhance retention regardless of timing. Our study shows researchers have options for how to incorporate incentives.

This study has several limitations. First, although we obtained several measures of demographic characteristics, additional parental characteristics may account for group differences. The demographic characteristics in our study predicting follow-up completion were similar to those in other cohort studies and longitudinal surveys [48–50] suggesting relevant measures were included. Second, this study was conducted with a sample of children treated at level 1 pediatric trauma centers for a serious or greater injury. These results may not generalize to studies of children with less severe injuries or those treated in other care settings. The results may also not apply to adults or patients with other conditions. Our findings should be confirmed in other populations to evaluate generalizability. Third, our experiment did not include a no-incentive condition. Without this baseline for comparison, we could not assess how incentives encouraged survey completion regardless of timing. Because the study was closed to enrollment earlier than anticipated, we were unable to reach our originally targeted sample size.

## Conclusions

This study assessed whether a prepaid incentive could improve retention in a longitudinal health outcomes study. Our approach provides a framework to apply and evaluate other response-inducing techniques from survey research in prospective studies. Because incentive timing did not affect retention in this study of injured children, researchers can choose either option for similar studies. Additional investigation is needed to identify methods that improve participation among underrepresented socioeconomic and ethnic subgroups. Without adequate representation, the conclusions drawn from health outcomes research may miss insights that are critical for addressing health disparities.

## Supplementary Information

**Additional file 1.** CONSORT Flow Diagram

**Additional file 2.** Children’s injury information, overall study sample and by experimental arm

**Additional file 3.** Demographics by follow-up survey completion status and experimental arm

## Data Availability

The datasets analyzed in the current study will be made available in a public-use data repository.

## Abbreviations

CPCCRN: Collaborative Pediatric Critical Care Research Network
FSS: Functional Status Scale
REDCap: Research Electronic Data Capture
Q1: 25^th^ percentile
Q3: 75^th^ percentile

## References

[1] National Research Council (2013). Nonresponse in social science surveys: a research agenda.

[2] Galea S, Tracy M (2007). Participation rates in epidemiologic studies. Ann Epidemiol..

[3] Marcellus L (2004). Are we missing anything? Pursuing research on attrition. Can J Nurs Res..

[4] Gustavson K, von Soest T, Karevold E, Røysamb E (2012). Attrition and generalizability in longitudinal studies: findings from a 15-year population-based study and a Monte Carlo simulation study. BMC Public Health..

[5] McDonald B, Haardoerfer R, Windle M, Goodman M, Berg C (2017). Implications of attrition in a longitudinal web-based survey: an examination of college students participating in a tobacco use study. JMIR Public Health Surveill..

[6] Brilleman SL, Pachana NA, Dobson AJ (2010). The impact of attrition on the representativeness of cohort studies of older people. BMC Med Res Methodol..

[7] Watson N, Wooden M, Lynn P (2009). Identifying factors affecting longitudinal survey response. Methodology of longitudinal surveys.

[8] Satherley N, Milojev P, Greaves LM, Huang Y, Osborne D, Bulbulia J, Sibley CG (2015). Demographic and psychological predictors of panel attrition: evidence from the New Zealand attitudes and values study. PLoS ONE..

[9] Brueton VC, Tierney JF, Stenning S, Meredith S, Harding S, Nazareth I, Rait G (2014). Strategies to improve retention in randomised trials: A Cochrane systematic review and meta-analysis. BMJ Open..

[10] Teague S, Youssef GJ, Macdonald JA, Sciberras E, Shatte A, Fuller-Tyszkiewicz M, Greenwood C, McIntosh J, Olsson CA, Hutchinson D (2018). Retention strategies in longitudinal cohort studies: A systematic review and meta-analysis. BMC Med Res Methodol..

[11] Booker CL, Harding S, Benzeval M (2011). A systematic review of the effect of retention methods in population-based cohort studies. BMC Public Health..

[12] Robinson KA, Dinglas VD, Sukrithan V, Yalamanchilli R, Mendez-Tellez PA, Dennison-Himmelfarb C, Needham DM (2015). Updated systematic review identifies substantial number of retention strategies: using more strategies retains more study participants. J Clin Epidemiol..

[13] Morgan AJ, Rapee RM, Bayer JK (2017). Increasing response rates to follow-up questionnaires in health intervention research: randomized controlled trial of a gift card prize incentive. Clin Trials..

[14] Mercer A, Caporaso A, Cantor D, Townsend R (2015). How much gets you how much? Monetary incentives and response rates in household surveys. Public Opin Q..

[15] Edwards PJ, Roberts I, Clarke MJ, Diguiseppi C, Wentz R, Kwan I, Cooper R, Felix LM, Pratap S. Methods to increase response to postal and electronic questionnaires. Cochrane Database Syst Rev. 2009;(3):MR000008. 10.1002/14651858.MR000008.pub4. 10.1002/14651858.MR000008.pub4 PMC8941848 19588449

[16] Church AH (1993). Estimating the effect of incentives on mail survey response rates: a meta-analysis. Public Opin Q..

[17] Singer E, Ye C (2013). The use and effects of incentives in surveys. Ann Am Acad Pol Soc Sci..

[18] Fumagalli L, Laurie H, Lynn P (2013). Experiments with methods to reduce attrition in longitudinal surveys. J R Stat Soc Ser A Stat Soc..

[19] Laurie H, Lynn P, Lynn P (2009). The use of respondent incentives on longitudinal surveys. Methodology of longitudinal surveys.

[20] Kretschmer S, Muller G (2017). The wave 6 NEPS adult incentive experiment. Methoden Daten Anal..

[21] Young B, Bedford L, das Nair R, Gallant S, Littleford R, JFR R, Schembri S, Sullivan FM, Vedhara K, Kendrick D (2020). Unconditional and conditional monetary incentives to increase response to mailed questionnaires: a randomized controlled study within a trial (SWAT). J Eval Clin Pract..

[22] LaRose R, Tsai H-YS (2014). Completion rates and non-response error in online surveys: Comparing sweepstakes and pre-paid cash incentives in studies of online behavior. Comput Human Behav..

[23] Lesser VM, Dillman DA, Carlson J, Lorenz F, Mason R, Willits F (2001). Quantifying the influence of incentives on mail survey response rates and their effects on nonresponse error.

[24] Petrolia DR, Bhattacharjee S (2009). Revisiting incentive effects: evidence from a random-sample mail survey on consumer preferences for fuel ethanol. Public Opin Q..

[25] Parsons NL, Manierre MJ (2014). Investigating the relationship among prepaid token incentives, response rates, and nonresponse bias in a web survey. Field Method..

[26] Teixeira R, Queiroga AC, Freitas AI, Lorthe E, Santos AC, Moreira C, Barros H (2021). Completeness of retention data and determinants of attrition in birth cohorts of very preterm infants: a systematic review. Front Pediatr..

[27] Lipps O (2010). Effects of different incentives on attrition and fieldwork effort in telephone household panel surveys. Surv Res Methods..

[28] Becker R, Glauser D (2018). Are prepaid monetary incentives sufficient for reducing panel attrition and optimizing the response rate? An experiment in the context of a multi-wave panel with a sequential mixed-mode design. Bull Methodol Sociol..

[29] Pollack MM, Holubkov R, Glass P, Dean JM, Meert KL, Zimmerman J, Anand KJ, Carcillo J, Newth CJ, Harrison R (2009). Functional status scale: new pediatric outcome measure. Pediatrics..

[30] Burd RS, Jensen AR, VanBuren JM, et al. Factors Associated With Functional Impairment After Pediatric Injury. JAMA Surg. 2021;156(8):e212058. 10.1001/jamasurg.2021.2058. 10.1001/jamasurg.2021.2058 PMC8173466 34076684

[31] Harris PA, Taylor R, Minor BL, Elliott V, Fernandez M, O’Neal L, McLeod L, Delacqua G, Delacqua F, Kirby J (2019). The REDCap consortium: Building an international community of software platform partners. J Biomed Inform.

[32] Watson RS, Choong K, Colville G, Crow S, Dervan LA, Hopkins RO, Knoester H, Pollack MM, Rennick J, Curley MAQ (2018). Life after critical illness in children-toward an understanding of pediatric post-intensive care syndrome. J Pediatr..

[33] Pinto NP, Rhinesmith EW, Kim TY, Ladner PH, Pollack MM (2017). Long-term function after pediatric critical illness: results from the Survivor Outcomes Study. Pediatr Crit Care Med..

[34] Becker R, Möser S, Glauser D (2019). Cash vs. vouchers vs. gifts in web surveys of a mature panel study––main effects in a long-term incentives experiment across three panel waves. Soc Sci Res..

[35] Pace LE, Lee YS, Tung N, Hamilton JG, Gabriel C, Raja SC, Jenkins C, Braswell A, Domchek SM, Symecko H (2020). Comparison of up-front cash cards and checks as incentives for participation in a clinician survey: a study within a trial. BMC Med Res Methodol..

[36] Avdeyeva OA, Matland RE (2012). An experimental test of mail surveys as a tool for social inquiry in Russia. Int J Public Opin Res..

[37] Natale JE, Lebet R, Joseph JG, Ulysse C, Ascenzi J, Wypij D, Curley MAQ (2017). Racial and ethnic disparities in parental refusal of consent in a large, multisite pediatric critical care clinical trial. J Pediatr..

[38] Cui Z, Truesdale KP, Robinson TN, Pemberton V, French SA, Escarfuller J, Casey TL, Hotop AM, Matheson D, Pratt CA (2019). Recruitment strategies for predominantly low-income, multi-racial/ethnic children and parents to 3-year community-based intervention trials: Childhood obesity prevention and treatment research (coptr) consortium. Trials..

[39] George S, Duran N, Norris K (2014). A systematic review of barriers and facilitators to minority research participation among African Americans, Latinos, Asian Americans, and Pacific Islanders. Am J Public Health..

[40] Jang M, Vorderstrasse A (2019). Socioeconomic status and racial or ethnic differences in participation: Web-based survey. JMIR Res Protoc..

[41] Kaiser BL, Thomas GR, Bowers BJ (2017). A case study of engaging hard-to-reach participants in the research process: community advisors on research design and strategies (CARDS)®. Res Nurs Health..

[42] Paskett ED, Reeves KW, McLaughlin JM, Katz ML, McAlearney AS, Ruffin MT, Halbert CH, Merete C, Davis F, Gehlert S (2008). Recruitment of minority and underserved populations in the united states: The centers for population health and health disparities experience. Contemporary Clinical Trials..

[43] Varni JW, Limbers CA, Burwinkle TM (2007). Parent proxy-report of their children’s health-related quality of life: An analysis of 13,878 parents’ reliability and validity across age subgroups using the PedsQL 4.0 generic core scales. Health Qual Life Outcomes.

[44] Schor EL (2003). Family pediatrics: report of the task force on the family. Pediatrics..

[45] Hunt JR, White E (1998). Retaining and tracking cohort study members. Epidemiol Rev..

[46] Wiant K, Geisen E, Creel D, Willis G, Freedman A, de Moor J, Klabunde C (2018). Risks and rewards of using prepaid vs. Postpaid incentive checks on a survey of physicians. BMC Med Res Methodol.

[47] Dillman DA, Smyth JD, Christian LM (2014). Internet, phone, mail, and mixed-mode surveys: the tailored design method.

[48] Patel MX, Doku V, Tennakoon L (2003). Challenges in recruitment of research participants. Adv Psychiatr Treat..

[49] Wu V, Abo-Sido N, Espinola JA, Tierney CN, Tedesco KT, Sullivan AF, Camargo CA (2017). Predictors of successful telephone follow-up in a multicenter study of infants with severe bronchiolitis. Ann Epidemiol..

[50] Young AF, Powers JR, Bell SL (2006). Attrition in longitudinal studies: Who do you lose?. Aust N Z J Public Health..

