# Supplementary material for: Incentive delivery timing and follow-up survey completion in a prospective cohort study of injured children: a randomized experiment comparing prepaid and postpaid incentives
Source: BMC Med Res Methodol. 2021 Oct 27;21:233. doi: 10.1186/s12874-021-01421-8 (PMC8549144; doi:10.1186/s12874-021-01421-8)
Supplement: Supplementary file 1 — Additional file 1. CONSORT Flow Diagram [file 12874_2021_1421_MOESM1_ESM.docx]

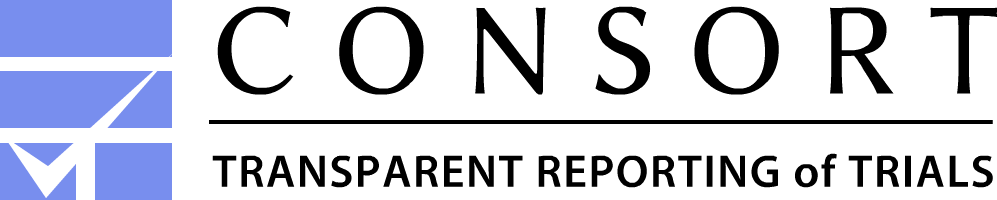


**CONSORT 2010 Flow Diagram**

Excluded (n= 415)

♦  Not meeting inclusion criteria (n= 182)

♦  Declined to participate (n= 65)

♦  Other reasons (n= 168)

## Analysis

Lost to follow-up (did not respond to survey) (n= 65)

Note: Non-responders included in analysis

Analysed (n= 204)
♦ Respondents (n= 139)

♦ Nonrespondents (n=65)

♦ Excluded from analysis (n= 0)

Analysed (n= 216)
♦ Respondents (n= 139)

♦ Nonrespondents (n=72)

♦ Excluded from analysis (n= 0)

Randomized (n= 420 parents)

Assessed for eligibility (n= 835 children)

## Follow-Up

Lost to follow-up (did not respond to survey) (n= 72)

Note: Non-responders included in analysis

## Enrollment

Allocated to prepaid incentive (n= 204 parents)

♦ Received allocated intervention (n=204)

♦ Did not receive allocated intervention (n= 0)

## Allocation

Allocated to postpaid incentive (n=216 parents)

♦ Received allocated intervention (n=216)

♦ Did not receive allocated intervention (n= 0)
