# Supplementary material for: Incentive delivery timing and follow-up survey completion in a prospective cohort study of injured children: a randomized experiment comparing prepaid and postpaid incentives
Source: BMC Med Res Methodol. 2021 Oct 27;21:233. doi: 10.1186/s12874-021-01421-8 (PMC8549144; doi:10.1186/s12874-021-01421-8)
Supplement: Supplementary file 2 — Additional file 2. Children’s injury information, overall study sample and by experimental arm [file 12874_2021_1421_MOESM2_ESM.docx]

**Additional File 2. Child’s injury information, overall study sample and by experimental arm**

|  | Incentive timing | |  |
| --- | --- | --- | --- |
|  | Before survey (N = 204) | After survey (N = 216) | Overall (N = 420) |
| Injury body region (n, %) |  |  |  |
| Multiple regions, with head (severe) | 7 (3.4) | 15 (6.9) | 22 (5.2) |
| Multiple regions, with head (not severe) | 5 (2.5) | 8 (3.7) | 13 (3.1) |
| Multiple regions, excluding head | 13 (6.4) | 23 (10.6) | 36 (8.6) |
| Isolated head (severe) | 14 (6.9) | 9 (4.2) | 23 (5.5) |
| Isolated head (not severe) | 42 (20.6) | 38 (17.6) | 80 (19.0) |
| Isolated thorax | 14 (6.9) | 16 (7.4) | 30 (7.1) |
| Isolated abdomen | 44 (21.6) | 37 (17.1) | 81 (19.3) |
| Isolated spine | 10 (4.9) | 11 (5.1) | 21 (5.0) |
| Isolated extremity | 55 (27.0) | 59 (27.3) | 114 (27.1) |
| Functional status scale (FSS)^2^ score at discharge (median [Q1, Q3] ^3^) | 6.0 [6.0, 7.0] | 7.0 [6.0, 8.0] | 7.0 [6.0, 7.0] |
| Change of ≥ 2 in any FSS domain from baseline to discharge (n, %) |  |  |  |
| No | 170 (83.3) | 177 (81.9) | 347 (82.6) |
| Yes | 34 (16.7) | 39 (18.1) | 73 (17.4) |
| Suspected child physical abuse (n, %) |  |  |  |
| No | 182 (89.2) | 194 (89.8) | 376 (89.5) |
| Yes | 22 (10.8) | 22 (10.2) | 44 (10.5) |
| Any chronic diagnoses (n, %) |  |  |  |
| No | 174 (85.3) | 180 (83.3) | 354 (84.3) |
| Yes | 30 (14.7) | 36 (16.7) | 66 (15.7) |
| Discharged to (n, %) |  |  |  |
| Home with parents/guardian | 180 (88.2) | 182 (84.3) | 362 (86.2) |
| Foster care | 4 (2.0) | 1 (0.5) | 5 (1.2) |
| Inpatient rehabilitation facility | 19 (9.3) | 26 (12.0) | 45 (10.7) |
| Other | 1 (0.5) | 6 (2.8) | 7 (1.7) |
| Unknown^4^ | 0 (0.0) | 1 (0.5) | 1 (0.2) |

^1^ P-value reported from logistic regression predicting incentive assignment controlling for site.

^2^ Pollack MM, Holubkov R, Glass P, Dean JM, Meert KL, Zimmerman J, Anand KJ, Carcillo J, Newth CJ, Harrison R *et al*. Functional Status Scale: New pediatric outcome measure. Pediatrics. 2009;124(1):e18-28

^3^ Q1, Q3: Interquartile range 25^th^ percentile and Interquartile range 75^th^ percentile.

^4^ Not included in the p-value calculation
