# Supplementary material for: Incentive delivery timing and follow-up survey completion in a prospective cohort study of injured children: a randomized experiment comparing prepaid and postpaid incentives
Source: BMC Med Res Methodol. 2021 Oct 27;21:233. doi: 10.1186/s12874-021-01421-8 (PMC8549144; doi:10.1186/s12874-021-01421-8)
Supplement: Supplementary file 3 — Additional file 3. Demographics by follow-up survey completion status and experimental arm [file 12874_2021_1421_MOESM3_ESM.docx]

**Additional File 3. Demographics by follow-up survey completion status and experimental arm**

|  | Before survey | | | After survey | |  |
| --- | --- | --- | --- | --- | --- | --- |
|  | Completed follow-up (N=139) | Did not complete follow-up (N=65) | Completed follow-up (N=144) | | Did not complete follow-up (N=72) | P-value^1^ |
| Age in years (median [Q1, Q3] ^2^) | 6.9 [2.9, 11.4] | 5.9 [2.8, 10.8] | 8.5 [2.6, 11.9] | | 6.8 [1.8, 12.2] | 0.83 |
| Female (n, %) | 54 (70.1) | 23 (29.9) | 45 (57.7) | | 33 (42.3) | 0.084 |
| Race (n, %) |  |  |  | |  | 0.91 |
| American Indian or Alaska Native | 0 (0.0) | 1 (100.0) | 1 (100.0) | | 0 (0.0) |  |
| Asian | 1 (50.0) | 1 (50.0) | 4 (80.0) | | 1 (20.0) |  |
| Black or African American | 22 (55.0) | 18 (45.0) | 32 (59.3) | | 22 (40.7) |  |
| White | 108 (72.5) | 41 (27.5) | 88 (71.5) | | 35 (28.5) |  |
| More than one | 7 (70.0) | 3 (30.0) | 16 (61.5) | | 10 (38.5) |  |
| Other | 1 (50.0) | 1 (50.0) | 2 (33.3) | | 4 (66.7) |  |
| Ethnicity (n, %) |  |  |  | |  | 0.67 |
| Hispanic or Latino | 8 (36.4) | 14 (63.6) | 10 (40.0) | | 15 (60.0) |  |
| Not Hispanic or Latino | 129 (71.7) | 51 (28.3) | 134 (70.2) | | 57 (29.8) |  |
| Unknown^3^ | 2 (100.0) | 0 (0.0) |  | |  |  |
| Parent preferred language-Spanish^4^ (n, %) | 0 (0.0) | 8 (100.0) | 1 (14.3) | | 6 (85.7) |  |
| Insurance (n, %) |  |  |  | |  | 0.78 |
| Private/commercial | 74 (78.7) | 20 (21.3) | 72 (77.4) | | 21 (22.6) |  |
| Medicaid/Medicare | 48 (53.9) | 41 (46.1) | 59 (56.2) | | 46 (43.8) |  |
| Self-Pay/no insurance | 6 (66.7) | 3 (33.3) | 2 (66.7) | | 1 (33.3) |  |
| More than one/other | 9 (90.0) | 1 (10.0) | 10 (76.9) | | 3 (23.1) |  |
| Unknown^3^ | 2 (100.0) | 0 (0.0) | 1 (50.0) | | 1 (50.0) |  |
| Annual household income (n, %) |  |  |  | |  | 0.38 |
| Less than $15,000 | 12 (37.5) | 20 (62.5) | 21 (61.8) | | 13 (38.2) |  |
| $15,000-$19,999 | 5 (62.5) | 3 (37.5) | 7 (53.8) | | 6 (46.2) |  |
| $20,000-$29,999 | 10 (66.7) | 5 (33.3) | 11 (47.8) | | 12 (52.2) |  |
| $30,000-$39,999 | 8 (66.7) | 4 (33.3) | 14 (66.7) | | 7 (33.3) |  |
| $40,000-$49,999 | 11 (61.1) | 7 (38.9) | 10 (52.6) | | 9 (47.4) |  |
| $50,000-$74,999 | 22 (88.0) | 3 (12.0) | 20 (76.9) | | 6 (23.1) |  |
| ≥ $75,000 | 60 (82.2) | 13 (17.8) | 55 (79.7) | | 14 (20.3) |  |
| Unknown^3^ | 11 (52.4) | 10 (47.6) | 6 (54.5) | | 5 (45.5) |  |
| Primary caregiver education (n, %) |  |  |  | |  | 0.68 |
| High school diploma/GED or less | 37 (52.9) | 33 (47.1) | 41 (51.3) | | 39 (48.8) |  |
| Associates/vocational degree/some college | 33 (71.7) | 13 (28.3) | 46 (70.8) | | 19 (29.2) |  |
| Bachelor's degree | 31 (77.5) | 9 (22.5) | 36 (87.8) | | 5 (12.2) |  |
| Graduate degree | 30 (81.1) | 7 (18.9) | 19 (79.2) | | 5 (20.8) |  |
| Unknown^3^ | 8 (72.7) | 3 (27.3) | 2 (33.3) | | 4 (66.7) |  |

^1^ P-value is interaction term p-value from logistic regression model predicting follow-up survey completion status with site, variable, incentive group, and interaction between variable and incentive group as predictors.

^2^ Q1, Q3: Interquartile range 25^th^ percentile and Interquartile range 75^th^ percentile.

^3^ Not included in P-value calculation.

^4^ P-value is not calculable for preferred language because of separation issues with the model due to no Spanish speakers assigned to incentive before survey completing follow-up.
